# Freezing Medium Containing 5% DMSO Enhances the Cell Viability and Recovery Rate After Cryopreservation of Regulatory T Cell Products *ex vivo* and *in vivo*

**DOI:** 10.3389/fcell.2021.750286

**Published:** 2021-12-03

**Authors:** Daniel Kaiser, Natalie Maureen Otto, Oliver McCallion, Henrike Hoffmann, Ghazaleh Zarrinrad, Maik Stein, Carola Beier, Isabell Matz, Marleen Herschel, Joanna Hester, Guido Moll, Fadi Issa, Petra Reinke, Andy Roemhild

**Affiliations:** ^1^Berlin Center for Advanced Therapies (BeCAT), Charité – Universitätsmedizin Berlin, Berlin, Germany; ^2^Berlin Institute of Health (BIH) Center for Regenerative Therapies (BCRT), Charité – Universitätsmedizin Berlin, Berlin, Germany; ^3^Department of Nephrology and Internal Intensive Care Medicine, Charité – Universitätsmedizin Berlin, Berlin, Germany; ^4^Transplantation Research and Immunology Group, Nuffield Department of Surgical Sciences, John Radcliffe Hospital, University of Oxford, Oxford, United Kingdom

**Keywords:** cell therapy, regulatory T cells (Tregs), cryopreservation, freeze-thawing, freezing medium, cell recovery rate, cell viability

## Abstract

Cell therapies have significant therapeutic potential in diverse fields including regenerative medicine, transplantation tolerance, and autoimmunity. Within these fields, regulatory T cells (Treg) have been deployed to ameliorate aberrant immune responses with great success. However, translation of the cryopreservation strategies employed for other cell therapy products, such as effector T cell therapies, to Treg therapies has been challenging. The lack of an optimized cryopreservation strategy for Treg products presents a substantial obstacle to their broader application, particularly as administration of fresh cells limits the window available for sterility and functional assessment. In this study, we aimed to develop an optimized cryopreservation strategy for our CD4+CD25+Foxp3+ Treg clinical product. We investigate the effect of synthetic or organic cryoprotectants including different concentrations of DMSO on Treg recovery, viability, phenotype, cytokine production, suppressive capacity, and *in vivo* survival following GMP-compliant manufacture. We additionally assess the effect of adding the extracellular cryoprotectant polyethylene glycol (PEG), or priming cellular expression of heat shock proteins as strategies to improve viability. We find that cryopreservation in serum-free freezing medium supplemented with 10% human serum albumin and 5% DMSO facilitates improved Treg recovery and functionality and supports a reduced DMSO concentration in Treg cryopreservation protocols. This strategy may be easily incorporated into clinical manufacture protocols for future studies.

## Introduction

Adoptive and regulatory T cell (Treg) therapies offer promising new options for the treatment of various clinical indications originating from a compromised immune system (Bluestone and Tang, 2018; Raffin et al., 2019; Roemhild et al., 2020; Waldmann, 2021). Academic institutions recognized this potential early and were crucial in their development, moving these therapies through the clinical trial phases toward market authorization for this new class of “Advanced Therapy Medicinal Products” (ATMPs).

Regarding clinical translation, all these innovative approaches face the same regulatory and logistical challenges, some of which are new and still rapidly changing (Hickson et al., 2021). Regulatory requirements or their interpretation vary between major markets, such as North America and Europe, especially in the early clinical phases, but also between countries within Europe. Many requirements such as purity, potency, safety, and stability of ATMPs known from conventional drug manufacturing must be redefined and taken into consideration. Stability is particularly important if the products cannot be used immediately after manufacture. This is not only a major logistical problem, but also a safety issue, as microbiological testing can take up to 14 days, depending on the applied test. In addition to the safety aspect, product stability and storage also have a major impact on subsequent commercialization. The ability to manufacture “off the shelf” products helps to reduce manufacturing costs drastically thereby increasing the access to such therapies (Abou-El-Enein et al., 2016, 2017a,b; Fritsche et al., 2020).

The cell therapy based treatment of diseases after solid organ transplantation (SOT) or hematopoietic stem cell transplantation (HSCT) in conjunction with virus-specific effector T cell (Teff) products, often consisting of a mixture of CD4+ and CD8+ T cells, has rapidly evolved. These originated from autologous cell products with simple virus specificity to multi-virus specific allogeneic therapy options becoming accessible due to good outcomes with few side effects (Gerdemann et al., 2012). This is partly due to the amenability of these T cell products to freeze-thawing and cryostorage. In recent years, the potential of Tregs has been increasingly recognized, based on good tolerability and promising clinical data (Roemhild et al., 2020). For cryopreservation of cell products, such as Teff cells, a freezing medium based on 10% dimethyl-sulphoxide (DMSO) and 10% serum is commonly used. This standard medium did not yield satisfactory results for clinical grade Tregs in our hands.

Cryopreservation is a highly challenging process for cell therapy products that can substantially compromise their quality and clinical efficacy (Galipeau, 2013; Moll et al., 2014, 2016). Standard cryoprotectants such as DMSO, a low-molecular-weight agent that penetrates the cell membrane by forming pores and thus prevents intracellular ice formation by reducing the water content inside the cell (Mazur, 1984), can comprise concentration-dependent cell toxicity (Verheijen et al., 2019). Extracellular cytoprotective agents such as polyethylene glycol (PEG) have also been described (Moll et al., 2016). These have a higher molecular weight and reduce ice formation outside the cell by breaking the hydrogen bonds between molecules through spatial separation (Towey and Dougan, 2012). Interestingly, a number of cell types can protect themselves from thermal, oxidative, and osmotic stress by synthesizing so-called heat shock proteins (HSP; Lindquist and Craig, 1988). Heat shock proteins assume a wide variety of functions in this context, with anti-apoptotic, antioxidant and cytoprotective effects having been described (Concannon et al., 2003; Sharp et al., 2013; Shaik et al., 2017).

**FIGURE 1:**
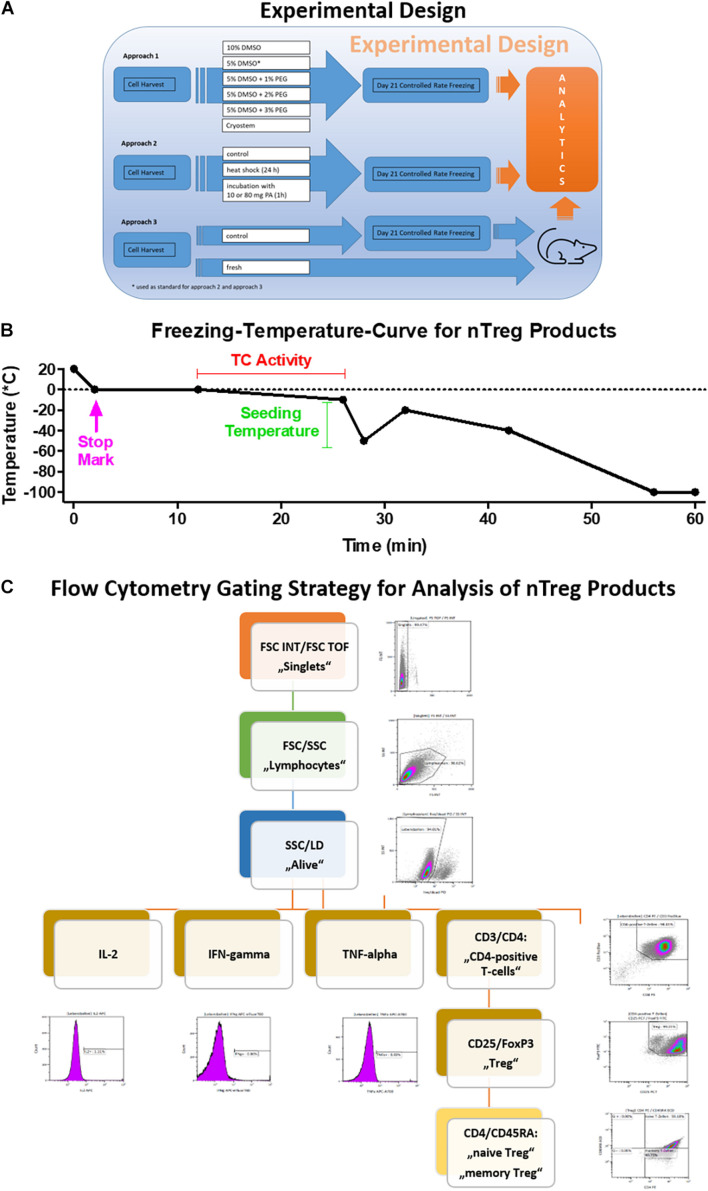
Freezing curve and flow cytometry gating strategy. **(A)** Experimental design and **(B)** Programmed freezing curve as shown in Tables 1, 2 [Stage Temp. (°C) Duration Heating Stop Seeding Temp. control] and **(C)** Gating scheme used for the analysis of the FACS data. The scheme ensures the exclusion of doublets and dead cells. It identifies lymphocytes first and uses the marker CD3 for the detection of CD4+ cells. The latter are further analyzed for their expression of the Treg relevant markers CD25 and Foxp3. The amount of cytokine producing cells is gated on all living lymphocytes.

Here, we use a manufacturing protocol based on the GMP-compliant production of our natural Treg (nTreg) cell product, as recently described in more detail in the report of our clinical study (Roemhild et al., 2020). Using freeze-thawing experiments, we assess cell product viability, the expression of characteristic cell surface markers (e.g., CD4/CD25/Foxp3), and cytokine secretion after stimulation. In addition to other tests usually employed for product release, such as sterility, endotoxin content, and mycoplasma testing, these parameters are of critical importance. We also assess the effects of lowering the DMSO concentration in the freezing medium on the above-mentioned parameters and critically, the product recovery rate post thawing, which is a decisive parameter, since it determines the number of viable cells available for therapy. For this purpose, freezing media with 5 and 10% DMSO content as well as a DMSO and serum-free synthetic cryoprotectant were compared. In addition, the optimization potential of a combination of intra- and extracellular cryoprotectant was investigated. Furthermore, *in vivo* survival of our fresh and thawed nTreg product was evaluated using an immunodeficient mouse model. Finally we tested the suppressive capacity of our Treg cell product after thawing. To our best knowledge, this is the first report on the effects of inducing cellular protective mechanisms in the context of cryopreservation of Treg cell products to achieve a storable product with reproducible and valid quality characteristics. The experimental design is shown in Figure 1A.

## Materials and Methods

For all experiments, 50 ml peripheral blood were collected from healthy volunteers or immunosuppressed patients after SOT. Peripheral blood mononuclear cells (PBMCs) were isolated by lymphopreparation density gradient centrifugation. The Charité Universitätsmedizin Berlin ethics committee and institutional review board (Hickson et al., 2021) approved the study protocol and all blood donors provided written informed consent.

### Manufacture of Natural Treg Cells

For isolation of CD8-CD25+ cells, PBMCs were isolated by density gradient centrifugation and MACS technology (Miltenyi Biotech) was used to deplete the CD8 cells from the PBMC fraction and then to enrich the CD25+ cells from the CD8-depleted cell fraction. CD4+CD25+ cells were first cultured in 96-hole round-bottom plates in X-Vivo 15 (Lonza) culture medium supplemented with 10% FBS (Biochrom), interleukin-2 (Novartis, 500 IU/ml), and rapamycin (Pfizer, 100 nM) at 37°C and 5% CO2. Polyclonal expansion was achieved by repetitive stimulation with anti-CD3/CD28 beads (MACS GMP ExpAct Treg Beads; Miltenyi Biotec) over a period of 21 days (Roemhild et al., 2020).

Depending on the cell density cells were transferred to 24-hole flat-bottom plates or for shortening the expansion time and to be closer to a GMP compliant “automated” and (semi)closed system to G-Rex bioreactors (Wilson Wolf) and continued to be cultured until harvest. For harvest, the cells were resuspended, washed several times, and expansion beads added during culture were depleted using MACS technology. In the first set of experiments to determine the DMSO concentration in the freezing medium and to optimize the medium with PEG, the Treg cell products were then cryopreserved directly. In the second set of experiments to optimize the cryopreservation strategy by activating cellular protective mechanisms, additional cell products were prepared. Cells were either bead depleted 1 day before harvest and cryopreservation and then incubated with two different concentrations of paeoniflorin (PA; Sigma) for 24 h, or exposed to a heat shock of 42°C for 1 h in a heating block on the day of harvest and cryopreservation.

### Composition of the Freezing Media

The composition of the different freezing media tested is shown in Table 1. Except for the Cryostem medium, which is synthetic and to be used without the addition of DMSO, the freezing medium consisted of the components dimethyl sulfoxide (DMSO), human serum albumin (HSA), and sodium chloride solution (NaCl). Shown are the media compositions of the investigated approaches, including the volumes of individual components calculated on 1.5 ml freezing medium. Moreover, the concentrations of polyethylene glycol (PEG) investigated in the optimization experiment as an additional supplement are given.

**TABLE 1:** Composition of freezing media.

**Approach**	**DMSO (%v/v)**	**DMSO (μl)**	**HSA (% v/v)**	**HSA (μl)**	**NaCl (% v/v)**	**NaCl (μl)**	**PEG (% v/v)**	**PEG (μl)**
1	5	75	10	750	85	675	–	–
2	10	150	10	750	80	600	–	–
3	Cryostem							
4	5	75	10	750	84	625	1	50
5	5	75	10	750	82	525	3	150
6	5	75	10	750	80	425	5	250

*The values are given as volume percent and calculated in μl per vial (assuming 1.5 ml content). Composition one and two served as standard for the two different main approaches.*

**TABLE 2:** Temperature ramps underlying the programmed freezing curve.

**Stage**	**Temp. (°C)**	**Duration (Wendering, #6)**	**Heating**	**Seeding**	**Temperature control**
1	0.0	3.0	Off		
2	0.0	10.0	Off		
3	–10.0	14.0	Off		active
4	–50.0	0.5	Off	active	
5	–20.0	5.0	On		
6	–35.0	10.0	Off		
7	–100.0	13.0	Off		
9	–100.0	2.0	Off		

*Within each ramp, the temperature is maintained for specified time before the program adjusts the temperature of the freezing chamber to the next stage. In stage 5, the heat generated by recrystallization during ice formation is counteracted for a short time (30 s) with a jump to lower temperature (−50°C).*

### Cryopreservation of Natural Treg Cells

All equipment/materials and buffers used for cryopreservation were precooled to 4°C. Subsequently, the washed and bead-depleted cells were divided into aliquots with identical cell concentrations. Depending on the experimental procedure, the cell pellet was resuspended in the appropriate cryopreservation medium and pipetted into cryotubes (1.5 ml). The cryotubes were then placed in the freezing machine (previously precooled to 4°C for 30 min). The Consarctic freezing machine with Biofreeze software then lowered the chamber temperature to −100°C in a controlled manner (duration approximately 55 min) (Figure 1B). During the freezing process, both the chamber temperature and the temperature of a reference tube filled only with the freezing medium were detected and recorded. After reaching a chamber temperature of −100°C, the samples were immediately transferred to a nitrogen tank and stored at <−150°C in the vapor phase until analysis.

### Thawing of Cryopreserved Natural Treg Cells and Analysis of Cell Number and Viability

To thaw the cryopreserved cells, a 50 ml tube was prepared with chilled (4–8°C) PBS (Biochrom) buffer containing 10% FBS. A cryotube containing 1.5 ml of cell suspension was then removed from the nitrogen storage container and swirled in a temperature-controlled water bath (37°C). Immediately after reaching a liquid phase, the cells were transferred into the prepared 50 ml tube under a sterile bench using a transfer pipette. After swirling the tube several times, the cells were pelleted in a precooled centrifuge (4°C) at 300 g for 10 min. Subsequently, the supernatant was removed and the pellet was resuspended in 15 ml PBS containing 10% FBS for further analyses. Dead and viable cells were determined using the CASY Cell Counter (OLS Life Science) based on the resistance measurement principle, as reported previously (Moll et al., 2014).

### Analysis of Treg Cell Phenotype and Effector Cytokine Production by FACS

Following harvesting or thawing of the nTreg cells, cell numbers were adjusted to 10 × 10^6^ cells/ml in stimulation-free medium (X-Vivo 15 with 10% FBS) and 500 μl of the cell suspension were pipetted into one FACS tube each and incubated at 37°C and 5% CO2 overnight. The next day, one of the two mixtures was stimulated with a PMA (Sigma Aldrich, 5 g/ml)-Inomycin (Sigma Aldric, 2.5 g/ml) solution and both tubes were incubated again for 1.5 h at 37°C and 5% CO2. Subsequently, 1 μl of Brefeldin-A (Sigma Aldrich) and 500 μl of stimulation-free medium were pipetted into each tube. This was followed by another incubation step for 4.5 h. After incubation, the cells were washed with cold PBS, centrifuged (10 min, 300 g, 4°C) and the cell number of both tubes was adjusted to 5 × 10^6^ cells/ml, and 100 μl of cell suspension from each of the two preparations was pipetted into three new FACS tubes each for antibody staining. This was followed by the addition of the anti-CD25 (Beckman Coulter, PC7), CD45RA (Beckman Coulter, ECD) and Live/Dead (Life Technologies, aqua 405 nm) antibody. After incubation of the samples for 30 min at 2–8°C, 500 μl of cold FixPerm buffer (eBioscience) was added to each tube. This was followed by centrifugation (10 min, 300 g, 4°C) and addition of FoxP3 (BD, FITC), CD4 (Beckman Coulter, PE), CD3 (Beckman Coulter, PacBlue), IFNγ (eBioscience, APC eFluor780), and IL-2 (BD, APC) antibodies onto the broken pellet. After a 30 min incubation at room temperature (RT) and another centrifugation step (10 min, 300 g, 4°C), the supernatant was discarded and pellets resuspended with 400 μl Perm buffer (eBioscience). This was followed by flow cytometric measurement using the Navios flow cytometer (Beckman Coulter). The flow cytometry gating is shown in Figure 1C.

### Treg Suppression Assay

Suppressive potency was examined as previously described (Wendering et al., 2019) CFSE-labeled autologous PBMCs were cultivated alone or in the prescence of different numbers of thawed corresponding Tregs. Seven PBMC Treg ratios ranging from 1:2 to 32:1 were analysed. αCD3/28 microbeads (Treg Suppression Inspector, Miltenyi Biotech) were applied for cell stimulation at a cell bead ratio of 1:1 (adjusted to the total cell number per well). After incubation at 37°C and 5% CO2 for 96 h cells were stained for live dead differentiation and CD3 surface antigen. Cell proliferation and its suppression was investigated by CFSE dilution. The percentage of proliferation suppression was calculated by relating the percentage of proliferating PBMCs in the prescence and absence of Tregs. Data were acquired on the Fortessa flow cytometer (BD) and analysed with FlowJo software (Treestar). Results are summarized in Figure 3C while FACS plots and calculation equation can be found in the Supplementary Figures 1,2.

### *In vivo* Analysis

Freshly expanded nTreg were manufactured at GMP facility in Berlin, transported in G-Rex (Wilson Wolf) culture chambers by overnight courier to the project partner (RESTORE) in United Kingdom and processed immediately upon arrival. Freshly expanded nTreg were transported in G-Rex (Wilson Wolf) culture chambers by overnight courier and processed immediately upon arrival. Cells were recovered into 50 mL Corning tubes and washed. Anti-CD3/CD28 beads (MACS GMP ExpAct Treg Beads; Miltenyi Biotec) were magnetically depleted (LS columns, Miltenyi Biotec). nTreg cryopreserved in 0.9% sodium chloride supplemented with 5% DMSO and 10% human serum albumin were transported by overnight courier in a temperature-controlled container on dry ice and, on arrival, were immediately transferred to a liquid nitrogen tank and stored at <−150°C in vapor phase. Thawing was achieved by gentle submersion in a pre-warmed 37°C water bath. On reaching a liquid phase, cells were transferred to a 50 mL Corning containing 30 μL DNAse and slowly diluted by intermittent swirling with pre-warmed (37°C) pure RPMI. Cells were centrifuged at 500 g for 5 min at room temperature and resuspended in pure RPMI. Viable cells were identified by 0.05% trypan blue exclusion with a haemocytometer under light microscopy. Immediately following recovery or thawing, nTreg were stained with violet proliferation dye (VPD, BD Biosciences) at a concentration of 1 μM (1 μL of 1 mM stock per mL) for 10 min in a 37°C water bath. 5 × 10^6^ VPD-labeled nTreg were injected into the peritoneal cavity of immunodeficient BALB/c Rag2^–/–^cγ^–/–^ mice. On day 6, cells were recovered by lavage of the peritoneal cavity with 10 mL room-temperature PBS. The following antibodies (fluorophore, manufacturer, clone) were used to stain recovered cells: anti-mouse CD45 (PE-eFluor^*TM*^ 610, Invitrogen, 30-F11) anti-human CD4 (APC, BD Pharmingen, 7137857), anti-human CD8 (APC-Cy7, BD Biosciences, SK1), anti-human CD25 (PE-Cy7, BD Biosciences, M-A251), anti-human CD3 (FITC, BioLegend, UCHT1). Dead cells were excluded with 7-AAD viability dye (Invitrogen, lot 2115592) Fluorescence was quantified by flow cytometry (BD FACSCanto, 3-laser).

## Results

### Recovery Rate and Cell Viability Benefit From Low DMSO Concentration in the Freezing Medium

**FIGURE 2:**
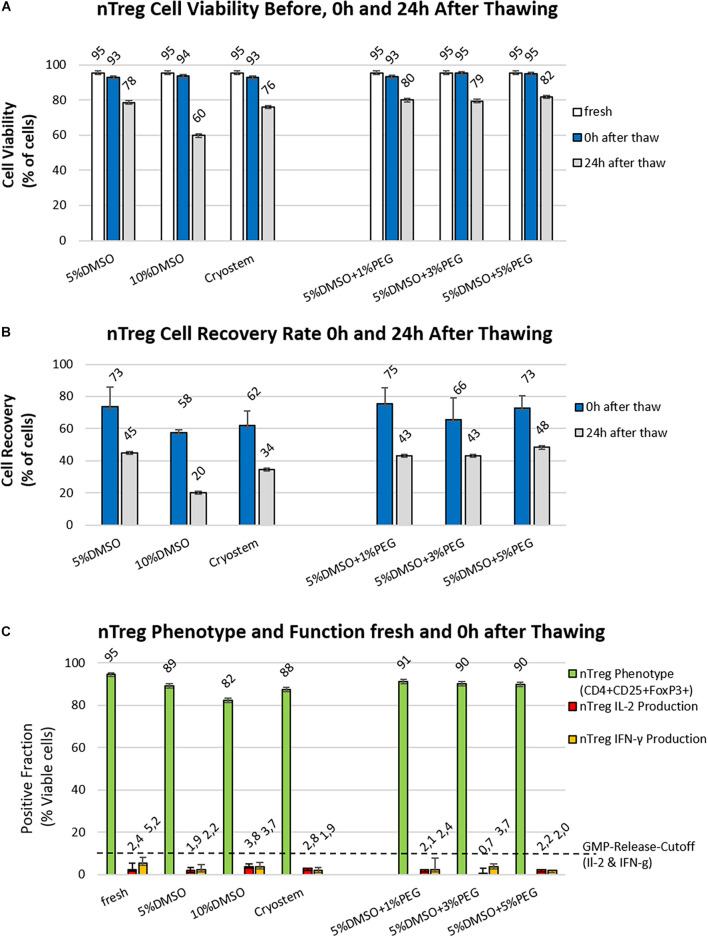
Optimizing nTreg cell viability and recovery post thawing. **(A,B)** Different readout parameters before freezing (white) and at 0 h (blue bars) or 24 h post thawing (gray bars) are shown for the six freezing media as listed in Table 1 (*n* = 2 cell products produced under GMP-like conditions). **(A)** Viability (%): There is only a small difference in viability between fresh cells and all the other preparations directly after thawing, but strong decreases are found at 24 h depending on the approach, freezing medium with 10% DMSO content performed the worst, while addition of PEG brings only minimal improvement in viability at 24 h compared to the freezing medium with 5% DMSO content; **(B)** Cell Recovery (%): Analysis at 24 h time point gives a more informative readout on quantitative differences and best performance with 5% DMSO; and **(C)** Phenotypic and Functional Analysis: Shown are the results of phenotyping as well as cytokine secreting cells before freezing (fresh) and after cryopreservation (thawed) for the investigated 6 different freezing media for the two initial nTreg cell products. The green bars show the percentage of Treg cells (% CD4+CD25+Foxp3+), the red bars represent the percentage of interleukin 2 (IL2)-producing cells, and the yellow bars show the percentage of interferon gamma (IFN-ɣ)-secreting cells both expressed as % positive cells.

Freezing media based on 10% DMSO and 10% serum are commonly used for cryopreservation of T cell products (Moll et al., 2016). We compared the standard freezing medium containing 10% DMSO with a freezing medium containing only 5% DMSO and synthetic Cryostem in a first set of experiments (Table 1). The recovery rate, as well as the viability of two Treg products were analyzed before freezing (fresh), immediately after thawing (0 h) and 1 day after thawing (24 h) (Figure 2A left panel). The cryopreservation process (Figure 1B) was associated with a substantial cell loss, indicated by poor recovery rates 0 h after thawing of 75–58% and 24 h after thawing of 48–20%. The viability of nTregs immediately after thawing was similar to the fresh cells before freezing (fresh 95 vs. 93–95% thawed) but decreased after an incubation of 24 h under culture conditions (5% DMSO: 78%, 10% DMSO: 60%, Cryostem: 76%). Freezing medium with 5% DMSO was superior to that with 10% DMSO content as well as the synthetic freezing medium in both recovery rate and viability.

### The Combination of DMSO and PEG as Freeze Protection Did Not Improve Recovery or Viability, While Treg Marker Expression Slightly Declined but Cytokine Secretion Was Not Affected

To optimize the cryopreservation medium, the superior medium composition containing 5% DMSO was supplemented with three different concentrations of PEG (1, 3, and 5%) in the second part of the experiment (Table 1 and Figure 2A right panel). However, these combinations of intracellular (DMSO) and extracellular (PEG) freeze protection did not improve recovery rate or cell viability compared to the medium with 5% DMSO without PEG addition. After a cryopreservation period of 35 days in the vapor phase of liquid nitrogen, clinical grade nTreg cell products from the two donors were thawed in a water bath at 37°C and compared with the cell product before freezing with respect to their phenotype (CD4+CD25+FoxP3+ cells of all CD4+ cells) and cytokine secretion (IL-2 and IFNγ) (Figure 2B), which serve as relevant release criteria for our clinical grade nTreg products. Duplicates of these cell products were stimulated with PMA/Iono or remained unstimulated and flow cytometry analysis was performed according to the gating scheme shown in Figure 1C. Compared to the fresh-from-culture-derived cells, the proportion of CD4+ and CD4+CD25+FoxP3+ within the “live-gated cells” decreased the most in the conventional freeze-thawing approach with 10% DMSO standard (95 vs. 82%). For the other approaches, the proportion decreased less (88 to 91%). The freezing medium with 5% DMSO content contained 89% CD4+CD25Foxp3+ cells after thawing. The proportion of cytokine-secreting cells did not change, which indicates no significant impact of cryopreservation on functional nTreg parameters (fresh vs. thawed IL2: 2.4 vs. 0.7–3.8% and IFNγ: 5.2 vs. 1.9–3.7%).

### Activation of Cellular Protective Mechanisms Before Cryopreservation Only Slightly Improved Cell Viability After Thawing With Only Minor Changes in Phenotype and Cytokine Secretion

**FIGURE 3:**
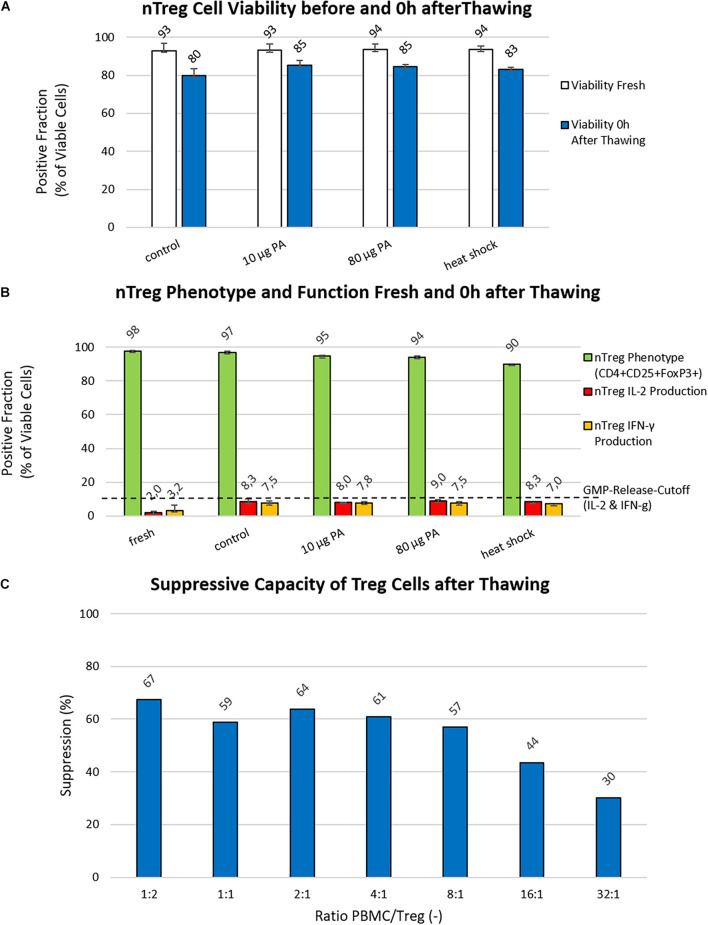
Analysis of Cryo-/Cyto-protective pathways and suppressive potency. **(A)** Viability (%): The viability of nTregs before freezing (white bars) and immediately after thawing/0 h (blue bars) is shown for the control approach (freezing medium with 5% DMSO) as well as the investigated approaches with heat shock or paeoniflorin (PA; 10 μg and 80 μg) induction of the cell’s own protective mechanisms. Data were collected using two generated Treg products from two different donors. **(B)** nTreg Phenotype and Function (%): The nTreg phenotype (green bars) and function assessed as the percentage of IL-2 (red bars) and IFN-g (yellow bars) cytokine secreting cells before freezing and after thawing of the two investigated nTreg cell products are shown. Depicted are the data of the cells before freezing, the control approach with 5% DMSO in the freezing medium (Control), the heat shock, and treatment with paeoniflorin (PA; 10 μg and 80 μg) induction. **(C)** Suppression Assay: The suppressive capacity of different PBMCs to Treg ratios are shown, starting with 1:2 PBMC:Treg down to 32:1.

Adding another cryoprotectant to the freezing medium did not yield an improvement in the first optimization attempt (Figure 2C). Thus, two additional nTreg products were manufactured to investigate the effect of inducing cellular heat shock mechanisms though a 24 h incubation with different concentrations of paeoniflorin (PA; 10 μg and 80 μg) on day 20 or a 1 h heat shock in a heating block at 42°C on day 21 (Figure 3A). The best performing freezing medium (5% DMSO content) was chosen as reference. With recovery rates comparable to the first set of experiments (data not shown), incubation with PA prior to cryopreservation increased cell viability immediately after thawing by a mean of 5.42% (10 μg) and 4.59% (80 μg), respectively. Heat shock increased this value by a mean of 3.11% compared to the control approach with pure freezing medium. The percentage of CD4+CD25+Foxp3+ cells was not substantially affected by cryopreservation compared with the reference value (5% DMSO without additives) (98 vs. 97%). For cellular protection induced by PA or heat shock, this percentage decreased by 3% (10 μg PA) and 8% (heat shock). Cytokine secretion increased slightly compared with fresh Treg cells for IL-2 (6–7%) and IFNγ (4–5%) in all approaches (Figure 3B). In comparison with the first two Treg products, the stable phenotype was confirmed when 5% DMSO was used as a cryoprotectant. The percentage of cytokine-secreting cells shows an opposite tendency. The percentage of cytokine secreting cells was slightly increased by cryopreservation compared to the comparative value before freezing. IL-2 producing cells increased from about 2% (fresh) to about 9% (after thawing) and the proportion of IFNγ-secreting cells increased from 1.5% (fresh) to approximately 6.5% (after thawing). All data from cryopreserved mixtures were in a similar range (IL-2: ±1.2%, IFNγ: ± 2.6%). Overall, however, the additional activation of cellular protective mechanisms does not seem to confer any advantage over the standard freezing medium supplemented with 5% DMSO.

### Freezing Medium With a Content of 5% DMSO Maintains the Suppressive Potential of Treg Cell Products After Cryopreservation

*In vitro* analysis of the suppressive capacity revealed that freezing medium with 5% DMSO content enables strong suppressive activity of cryopreserved Treg products after thawing. A 4-day proliferation assay showed that growth of effector cells is inhibited by approximately 60% (with a maximum of 67% at a ratio of 1:2) up to a ratio of 8 PBMC to 1 Treg cell. Figure 3C further shows that even at a ratio of 16 to 1, cell suppression is still approximately 43%.

### *In vivo* Survival of Natural Treg Is Comparable Between Freshly Produced and Cryopreserved Natural Treg Products

**FIGURE 4:**
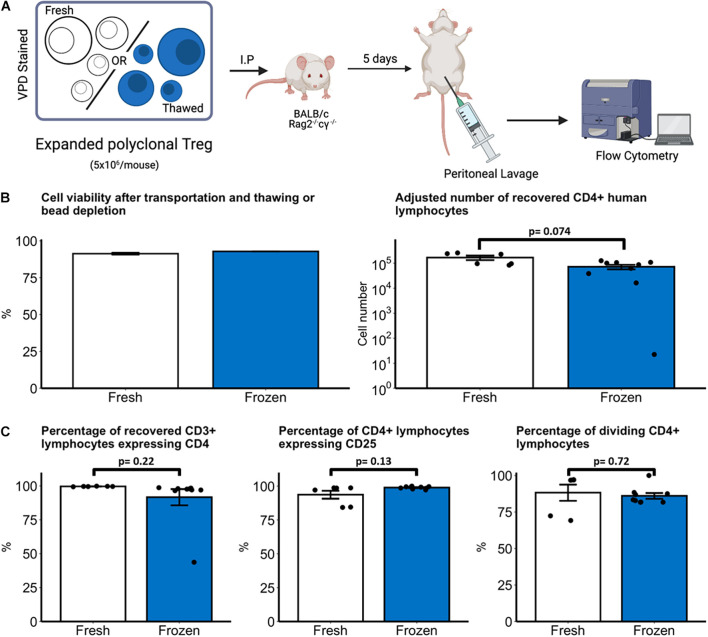
*In vivo* survival of fresh and thawed nTreg. **(A)** Experimental schematic, created with biorender.com. Freshly expanded or cryopreserved nTreg were recovered and stained with violet proliferation dye (VPD). Before injection into mice cells were characterized for Treg markers. VPD staining ensured the traceability to Treg-positive cells for later investigations. Immunodeficient BALB/c Rag2^– /–^cγ^– /–^ mice received 5 × 10^6^ VPD-labeled nTreg from one of two donors which were recovered after 5 days by peritoneal lavage **(B)**. Cell viability was quantified by light microscopy with 0.05% Trypan Blue dead-cell exclusion after transport and bead depletion (fresh nTreg) or transport and thawing (frozen nTreg). The number of nTreg recovered from lavage after 5 day incubation was quantified by flow cytometry. **(C)** Phenotype and proliferation of recovered human (mCD45^–^CD3^+^) lymphocytes were quantified by expression of CD4 and CD25. Division was defined as lymphocytes with VPD staining intensity less than the maximally stained (undivided) peak. Each point represents a separate mouse with *n* = 2 Treg donors. Data are represented as mean ± SEM and statistical significance determined using unpaired *t*-tests.

To ensure that freeze-thawing does not compromise *in vivo* survival, freshly expanded and cryopreserved nTreg were injected *via* the intraperitoneal route into immunodeficient BALB/c Rag2^–/–^cγ^–/–^ mice (5 × 10^6^/mouse) and recovered after 5 days (Figure 4A). We first assessed cell recovery of fresh or frozen cell products following overnight transportation and bead depletion. Cell viability was 91.3% (95% CI 90.0 – 92.7) compared with 92.8% viability following thawing (Figure 4B, left panel). Following cell transfer into mice, a mean of 16.9 × 10^4^ fresh and 8.8 × 10^4^ thawed nTreg were recovered after 5 days with no statistically significant difference between the groups (*p* = 0.074, Figure 4B, right panel). Within the human lymphocyte (mCD45-CD3+) population the phenotype between freshly expanded and thawed nTreg was assessed by expression of CD4 and CD25 (Figure 4C, left and middle panels). A mean of 99.7% fresh and 91.7% thawed nTreg expressed CD4. Expression of CD25 (IL2RA) within the CD4 population was also uniformly high across both populations with no statistically significant difference between the groups (*p* = 0.13). Finally, proliferation was assessed by categorizing proliferating and non-proliferating CD3+CD4+ lymphocytes based upon dilution of violet proliferation dye. Within the CD3+CD4+ population a mean of 88.2% fresh and 86.0% thawed nTreg underwent at least one division *in vivo* (Figure 4C, right panel) with no significant difference between the groups (*p* = 0.72).

## Discussion

Optimizing cryostorage and freeze-thawing procedures is an essential task for developing successful cell therapy products that can be incorporated into clinical practice (Galipeau, 2013; Moll et al., 2014, 2016). Here, we studied these procedures on four nTreg cell products prepared using our group’s GMP grade manufacturing process, which we have also applied to nTreg cell product generation during the ONE Study (Roemhild et al., 2020; Sawitzki et al., 2020). Our approach is based on the collection of a small volume of peripheral blood. To obtain sufficient cells for clinical application, the cells are subsequently expanded. To address the difficulties and limitations of freezing, expansion of thawed cells has been described (Golab et al., 2013), among others. Since from a regulatory point of view the expansion of frozen cells results in a new product to be released (EU GMP Guideline), this procedure is not suitable to be part of a manufacturing process. We first demonstrated that the cell recovery rate with all cryopreserved products, using 10% DMSO in freezing medium or synthetic Cryostem medium, was within the range of published data with approximately 60% recovery directly after thawing (Hippen et al., 2011; Golab et al., 2013; MacDonald et al., 2019). By using only 5% DMSO we were able to substantially increase the recovery rate directly (58 vs. 73%) and at 24 h (20 vs. 45%), as well as cell viability 24 h after thawing (60 vs. 78%).

When analyzing cell viability and recovery after thawing of therapeutic cells, the chosen analysis time point post-thawing has a major impact on the result, typically resulting in a further decline in cell viability and recovery 1 day post thawing (cells recovered in culture) compared with the viability obtained directly after thawing (Moll et al., 2014, 2016). Thus, when conducting this analysis after 24 h, the values for our analyzed nTregs dropped even further, suggesting irreversible cell damage due to cryopreservation or freeze-thawing (Christenson et al., 2012). Viability of recovered cells varied between 95% (first series of experiments) and 80% (second series of experiments) for the four nTreg products analyzed. This was not clear from other previous publications, which usually do not distinguish between viability and recovery rate, or offset both values against each other (MacDonald et al., 2019). The viability of nTreg cells decreased further 24 h after thawing, highlighting the negative influence of known factors such as DMSO cell toxicity or osmotic shock in the context of cryopreservation (Elkord, 2009; MacDonald et al., 2019). The standard freezing medium widely used for cryopreservation of a wide variety of cell types is based on a DMSO content of 10% (Broxmeyer et al., 2003). We have found that lowering the DMSO concentration had a positive effect on the result of Treg cryopreservation. The same has been reported for cryopreservation of cord blood cells (Hayakawa et al., 2010). Among others, a possible explanation might be a mitigation of the induction of drastic changes in human cellular processes by DMSO (Verheijen et al., 2019). In addition, Treg cells are known to have a different metabolic profile compared with Teff cells (Macintyre et al., 2014; Gerriets et al., 2015; Angelin et al., 2017; Hashimoto et al., 2020). Thus, one can speculate whether Treg cells, due to their different metabolism, are more susceptible to toxic effects when metabolizing DMSO. This could explain why, despite the improvement by 5% DMSO-containing medium, the results are 30% below the recovery of our CMV- or EBV-specific Teff products (data not shown).

To further improve the cryopreservation results of our expanded Treg cells, we first assessed the effects of supplementation of the cryoprotective effect of DMSO as a penetrating agent with polyethylene glycol (PEG) as an extracellular cryoprotectant. However, considering future commercialization of production, the results obtained are disproportionate to the added effort, due to the waxy consistency of pure PEG, which greatly impairs handling. In the next round of experiments, we tested whether activation of cellular protective mechanisms improves the cryopreservation process through a mechanism of action which has already been demonstrated to be beneficial in stem cell cryopreservation (Shaik et al., 2017). Expression of the protective heat shock proteins or heat shock factors was induced either thermally (heat shock at 42°C for 1 h) or by incubation with paeoniflorin (Sawitzki et al., 2020) before cryopreservation. This increased the viability directly after thawing by around 5% compared to cryomedium with 5% DMSO. Nevertheless, this in turn was offset by a considerable additional process-technical effort.

The scientific literature proves that Treg cell products with suppressive properties are generated when appropriate isolation techniques and culture medium containing rapamycin are applied. However, applying a proliferation assay, we also clearly demonstrated/confirmed/verified that the functional suppressive potency of the cell products is not negatively affected by cryopreservation or freeze-thawing with 5% DMSO-containing freezing medium. This is in line with various publications showing that the suppressive activity of Tregs is not significantly affected by the freeze-thaw processes (e.g., Nadig et al., 2010).

Our comparision of freshly expanded and cryopreserved nTreg recovery and phenotype, however, supports the application of cryopreserved nTreg products. In our mouse model, there were no differences in recovery, phenotype, or proliferation between fresh or thawed nTreg. We did observe one outlier animal within the thawed group from which few human cells were recovered, reducing the mean values of both recovered lymphocytes and the proportion of CD3+ lymphocytes expressing CD4+ within the frozen group. This may be related to technical factors which are unclear, such as failure to inject cells correctly into the peritoneum. The number of nTreg recovered by lavage is also in keeping with our previous experience with this model (Zaitsu et al., 2017). In clinical studies, Treg dosage is closely related to the eventual number of circulating Tregs (Harden et al., 2021), which is ultimately crucial for a therapeutic outcome (Tang and Lee, 2012). The clear practical advantages of ATMP cryopreservation for transportation and administration, in conjunction with the comparable *in vivo* survival data demonstrated here, supports the validity of cryopreservation and thawing as an approach to delivering nTreg cell therapy.

There are some limitations to this study. As experiments were conducted under GMP-like conditions with the associated high costs and other practical limitations, the number of nTreg products that could be studied here was limited (*n* = 2–4 depending on the experiment), thus more subtle changes over a large sample size have not been explored here. A consequence of this limitation is that no valid calculation of a correlation is possible. A number of other factors would be helpful to investigate in the future. From our perspective and in analogy to the developments observed with other cellular therapeutics (Galipeau, 2013; Moll et al., 2014, 2016, 2020; Hoogduijn et al., 2016), understanding the influence of cryopreservation on clinical grade nTreg products is critical for increasing their availability and safety. More in-depth studies are therefore required to assess metabolic characteristics of Tregs in the context of cryopreservation. In addition, knowledge surrounding applied procedures for freezing (e.g., metabolic reduction by cooling) and thawing (e.g., “uncontrolled” in a 37°C water bath) is crucial. While this study provides important reassurance regarding the use of thawed cryopreserved Treg cells, further work must focus on optimising all other procedures in the freeze-thaw process to ensure the maximum number of viable and functional cells eventually reaches the patient.

## Conclusion

In summary, we here demonstrate that reducing the amount of DMSO in the freezing medium from 10 to 5% improves the cell recovery rate and viability without negatively affecting the suppressive potential or our release criteria, such as relevant phenotypic markers or cytokine secretion profiles of our GMP Treg cell products. Furthermore, we investigated the combination of an intracellular and extracellular cryoprotectant and, to our knowledge, for the first time, the induction of cellular cyto-/cryo-protective mechanisms for clinical grade nTreg products. Unfortunately, no obvious larger optimization potential for cryopreservation has emerged from these pilot study approaches. Nonetheless, these studies involved substantial resources and time, due to simulation of the approaches within a GMP environment. Currently, the hurdles of unsatisfactory cryopreservation of nTregs still stand in the way of broader availability, the establishment of “off the shelf” approaches, and successful commercialization. Although the administration of fresh products is currently accepted by regulatory agencies in early-stage trials and clinical therapeutic results are promising, improved performance of clinical-grade nTreg products in cryostorage and freeze-thawing approaches would greatly facilitate their broader use. Although the focus of the many academically driven approaches in the cell therapy field is often on the isolation and expansion of specific cell populations, storage and clinical handling is an important central component of the manufacturing process. Thus, future efforts on cell therapy optimization, for nTregs and other cellular therapeutics alike, such as optimal storage, clinical handling and delivery to patients, should be an integral part of their development.

## Data Availability Statement

The raw data supporting the conclusions of this article will be made available by the authors, without undue reservation.

## Ethics Statement

The animal study was reviewed and approved by all mouse experiments were performed using protocols approved by the Committee on Animal Care and Ethical Review at the University of Oxford and in accordance with the United Kingdom Animals (Scientific Procedures) Act 1986 and under PPL number P8869535A.

## Author Contributions

DK, NO, OM, FI, GM, PR, and AR contributed in writing, review and editing, and conceptualization and supervision. DK, OM, HH, GZ, MS, CB, IM, MH, JH, and AR contributed in experiments and investigation. All authors contributed to the article and approved the submitted version.

## Conflict of Interest

The authors declare that the research was conducted in the absence of any commercial or financial relationships that could be construed as a potential conflict of interest.

## Publisher’s Note

All claims expressed in this article are solely those of the authors and do not necessarily represent those of their affiliated organizations, or those of the publisher, the editors and the reviewers. Any product that may be evaluated in this article, or claim that may be made by its manufacturer, is not guaranteed or endorsed by the publisher.
