# Supplementary figures and images for: Freezing Medium Containing 5% DMSO Enhances the Cell Viability and Recovery Rate After Cryopreservation of Regulatory T Cell Products ex vivo and in vivo
Source: Front Cell Dev Biol. 2021 Dec 3;9:750286. doi: 10.3389/fcell.2021.750286 (PMC8677839; doi:10.3389/fcell.2021.750286)

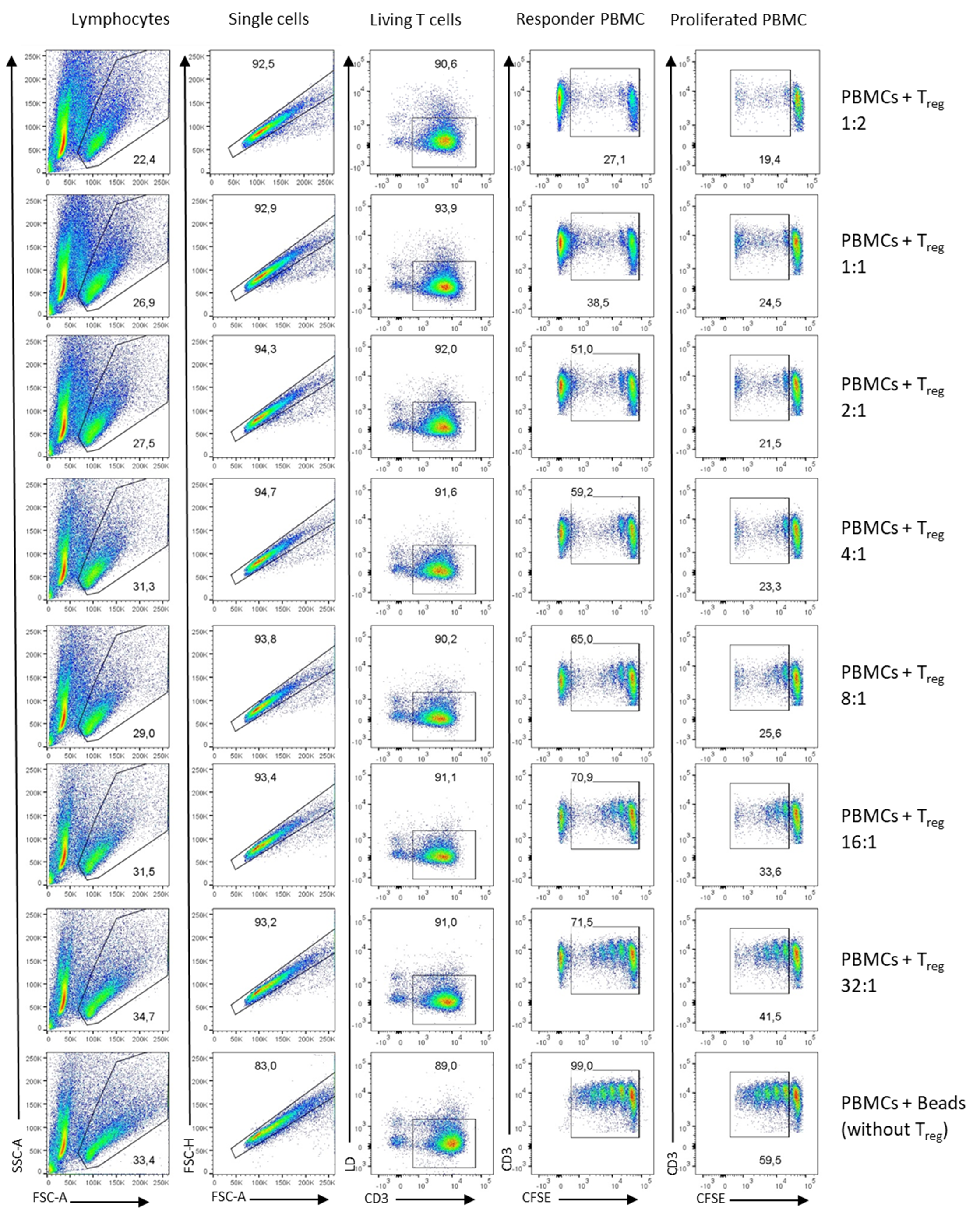

Supplement: Supplementary file 1 [file Image_1.tif]

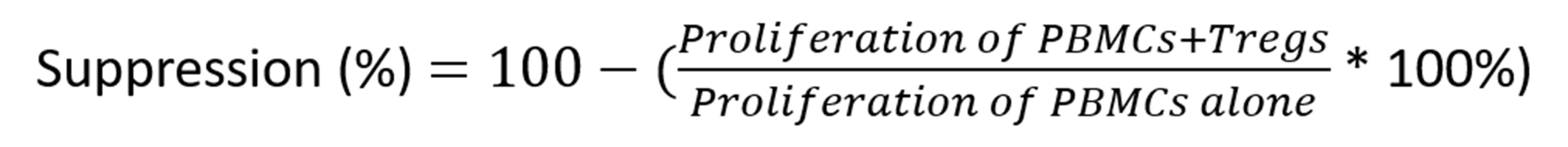

Supplement: Supplementary file 2 [file Image_2.tif]
